# Regulation of the Fanconi Anemia DNA Repair Pathway by Phosphorylation and Monoubiquitination

**DOI:** 10.3390/genes12111763

**Published:** 2021-11-05

**Authors:** Masamichi Ishiai

**Affiliations:** Central Radioisotope Division, National Cancer Center Research Institute, Tsukiji, Chuo-ku, Tokyo 104-0045, Japan; mishiai@ncc.go.jp; Tel.: +81-3-3542-2511 (ext. 3224)

**Keywords:** Fanconi anemia, ubiquitination, phosphorylation, ATR, ATM, interstrand crosslink, DNA repair

## Abstract

The Fanconi anemia (FA) DNA repair pathway coordinates a faithful repair mechanism for stalled DNA replication forks caused by factors such as DNA interstrand crosslinks (ICLs) or replication stress. An important role of FA pathway activation is initiated by monoubiquitination of FANCD2 and its binding partner of FANCI, which is regulated by the ATM-related kinase, ATR. Therefore, regulation of the FA pathway is a good example of the contribution of ATR to genome stability. In this short review, we summarize the knowledge accumulated over the years regarding how the FA pathway is activated via phosphorylation and monoubiquitination.

## 1. Introduction

Fanconi anemia (FA) is a rare human genetic disorder characterized by bone marrow failure, skeletal malformation, and increased incidence of cancer. Patients with FA carry inherited mutations in one of 22 FA genes (FANCA to FANCW) [1,2,3,4,5,6,7], which encode proteins constituting the FA pathway, also called the FA/BRCA pathway, which is a fundamental DNA repair mechanism that functions in the detection and repair of, as well as tolerance to, endogenous DNA damage. Cells from FA patients display elevated chromosome abnormalities and are hypersensitive to DNA interstrand crosslink (ICL)-inducing reagents, such as mitomycin C (MMC) and cisplatin. The role and function of the FA pathway was mainly revealed by the study of ICL repair [1,2,3,4].

**Figure 1:**
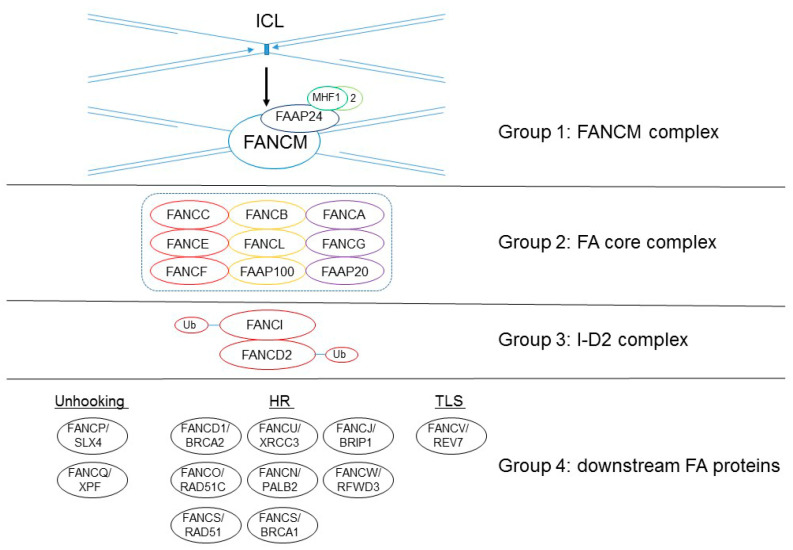
The 22 FANC proteins are classified into four groups according to their proposed functions in ICL repair. Recent cryo-electron microscopy (EM) studies have determined the fine structure of the FA core complex (group 2), which comprises nine proteins organized in three subcomplexes: BL100 (composed of two copies of FANCB, FANCL, and FAAP100), CEF (composed of a single copy of FANCC, FANCE, and FANCF), and AG20 (composed of two copies of FANCA and FANCG and 1 or 2 copies of FAAP100) [12,13,14,15]. Unhooking: ICL removal by cutting the activity of the nucleases.

These 22 FA gene products are classified into four functional groups, as follows: a damage-sensing module (group 1); a large E3 ubiquitin ligase, called the FA core complex (group 2); the key effector of FA pathway and the E3 ligase substrate the FANCI–FANCD2 (I-D2) heterodimer complex (group 3); and a large group of DNA repair/damage-tolerance factors (group 4) (Figure 1; [4,5]). The first group is the heterotetrametric FANCM complex, which is composed of the FANCM ATPase and its three interacting partners, FA-associated protein (FAAP) 24 and the histone hold proteins MHF1 (FAAP16/CENP-S) and MHF2 (FANP10/CENP-X). This FANCM complex recognizes stressed replication forks and helps recruit group 2, an E3 ubiquitin ligase of the FA core complex (this enzyme’s activity is carried out by FANCL, RING E3 ligase). The FA core complex is composed of FANCA, FANCB, FANCC, FANCE, FANCF, FANCG, FANCL, FAAP20, and FAAP100. The FA core complex recruits FANCT/UBE2T, an E2 ubiquitin-conjugating enzyme that monoubiquitinates the group 3 of the I-D2 complex. The monoubiqutinated I-D2 complex localizes at the DNA-damaged sites and interacts with other DNA-repair proteins, including the downstream group 4 FA proteins (FANCD1/BRCA2, FANCJ/BRIP1, FANCN/PALB2, FANCO/RAD51C, FANCR/RAD51, FANCS/BRCA1, FANCU/XRCC2, FANCQ/XPF, FANCP/SLX4, FANCV/REV7, and FANCW/RFWD3), enabling the removal of ICL, or performs DNA repair such as homologous recombination (HR) or translesion DNA synthesis (TLS) [1,2,3,4,5,6,7].

In addition to its role in ICL repair, FA proteins are also responsible for the maintenance of genome stability following replication stress from a variety of sources, including endogenous stress resulting from oncogenes or aldehyde accumulation; DNA damaging agents that disrupt replication, such as hydroxyurea; and a low-dose treatment of DNA polymerase inhibitors, such as aphidicolin [1,2,3,4,5]. FANCD2 and FANCI are required for the maintenance of two common fragile sites’ (CFSs) loci, FRA3B and FRA16D, in which the large tumor suppressor genes *FHIT* and *WWOX* reside. Recently, monoubiquitinated FANCD2 and FANCI have been accumulated in the CFS loci under mild replication stress in association with the formation of R-loop, where FANCD2 is required for R-loop resolution. The R-loop consists of a DNA–RNA hybrid and displaces single-stranded DNA, constituting a major threat to genome stability [8,9,10,11]. Collectively, these data reveal a comprehensive role of FA in the maintenance of genome integrity.

## 2. FANCD2 Monoubiquitination Is Key to Its Function in the FA Pathway

FANCD2 is critical for ICL repair. Monoubiquitination of FANCD2 is a landmark of FA pathway activation. FANCD2 is monoubiquitinated in vivo in response to DNA damage or replication stress in a manner dependent on the FA core complex, ATR-ATRIP kinase, FANCI, and E2 enzyme FANCT/UBE2T [1,2,3,4,5,16]. FANCD2 carrying a mutation of the monoubiquitination site lysine (K561 in human protein) can neither form foci nor relocalize to chromatin, and the exogenously expressed mutant protein cannot reverse the ICL sensitivity of FANCD2-deficient cells. The functional significance of FANCD2 monoubiquitination was addressed by using chicken FANCD2 carrying a mutated monoubiquitination site (K563R) fused with ubiquitin (D2KR-Ub) [17]. The expression of D2KR-Ub was able to reverse the ICL sensitivity of FANCD2-deficient cells to near-wild-type levels. In these cells, the D2KR-Ub protein could be found constitutively in chromatin; however, post-MMC foci formation could not be detected [17]. These phenotypes may resemble de-ubiquitination enzyme USP1 deficient cells, in which constitutive FANCD2 ubiquitination, loss of FANCD2 foci, and mild ICL sensitivity have been observed [16].

## 3. FANCI Phosphorylation Is a Molecular Switch Triggering FANCD2 and FANCI Monoubiquitination

**Figure 2:**
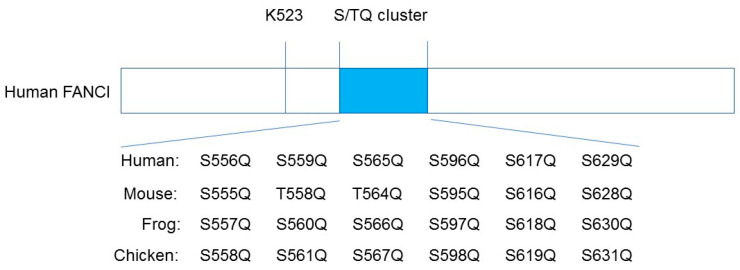
Schematic diagram of human FANCI, indicating the six putative phosphorylation sites within the S/TQ cluster domain. K523 is the monoubiquitination site of the human FANCI.

It has been proposed that ATR-ATRIP-mediated phosphorylation of six sites on FANCI, which is known as the S/TQ cluster, is critical for the monoubiquitination of I-D2, mostly based on the evidence that the substitution of alanine at all six clustered S/TQ sites near the monoubiquitination residue (the non-phosphorylatable mutant Ax6) abrogates FANCD2 ubiquitination (Figure 2; [18,19,20,21]). In addition, the phosphomimic mutant Dx6 (six aspartic acid replacements of the same residues as Ax6) induces constitutive monoubiquitination of FANCD2 in a chicken DT40 FANCI knockout cell line [18]. These studies have relied on electrophoresis-based methods (the phos-tag reagents); thus, the sites of the FANCI S/TQ cluster actually phosphorylated in vivo could not be determined.

A reconstituted in vitro I-D2 monoubiqutination and deubiquitination using purified proteins has been advanced in this decade, using the FANCL protein alone in the whole FA core complex as an E3 ligase [22,23,24,25,26]. FANCD2 monoubiqutination is greatly stimulated by the addition of FANCI and DNA [22,23]. For deubiquitination, USP1-UAF1 could efficiently remove ubiquitin from FANCD2-^Ubi^–FANCI, but not FANCD2-^Ubi^–FANCI-^Ubi^ [21]. When FANCD2-^Ubi^–FANCI-^Ubi^ is dissociated from the DNA, USP1-UAF1 removes ubiquitin from FANCD2-^Ubi^–FANCI-^Ubi^ [25]. Thus, functional significance must exist between FANCD2-^Ubi^–FANCI-^Ubi^ and FANCD2-^Ubi^–FANCI on DNA (see below).

## 4. Re-Evaluation of FANCI Phosphorylation in the Regulation of FA Pathway

Cheung et al. developed phospho-specific antibodies to three sites (S556, S559, and S565) in the human FANCI S/TQ cluster [27]. Consistent with previous reports, S556, S559, and S565 within the S/TQ cluster of human FANCI and equivalent residues of Frog (*Xenopus*) FANCI are indeed phosphorylated when analyzed by mass spectrometry and phosphor-specific antibodies against these three different sites. In contrast, the phosphorylation of S596, S617, and S629 of human FANCI and equivalent *Xenopus* residues (i.e., the remaining three residues within the S/TQ cluster) are not observed in mass spectrometry studies (Figure 2; [18,27,28]). This observation is consistent with studies on the crystal structure of the mouse I-D2 complex; three phosphorylated S556, S559, and S565 equivalent mouse sites exist on the surface of the I-D2 complex. On the other hand, non-phosphorylated S596, S617, and S629 equivalent mouse sites are buried in the structure of FANCI and are unlikely to be accessible to any kinases [21].

Tan et al. reported that using their reconstituted purified protein systems, the phosphatase-treated I-D2 complex eliminated the monoubiqutination of FANCI and FANCD2, but the addition of recombinant ATR-ATRIP kinase restored the monoubiqutnation level of both proteins [28]. Thus, these three residues are the substrates of ATR kinase, and are required for optimal monoubiquitination of FANCI and FANCD2. They also observed that ATR phosphorylation of FANCI specifically mediates its effects on FANCI, even when it is not bound to FANCD2 [28].

**Figure 3:**
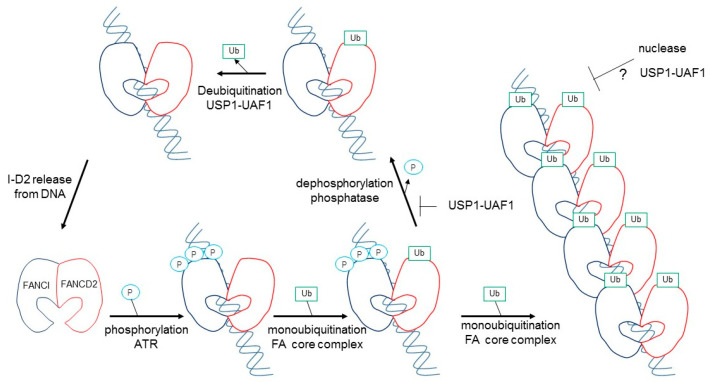
Proposed activation model of the FANCD2–FANCI complex by phosphorylation and monoubiquitination. See details in the text. This model was constructed according to the results of Tan et al. [28,30].

Tan et al. also evaluated the significance of these three phosphorylation sites using recombinant *Xenopus* FANCI and FANCD2 proteins. Phosphomimic mutations in S560 (S559 in human) together with S566 (S565 in human) are sufficient to maximally stimulate monoubiquitination of the I-D2 complex by the FA core complex [28]. In this regard, it was reported previously that *Xenopus* FANCI phosphorylation leads to the dissociation of the xI-D2 heterodimers, in comparison with the results from xFANCI-S6D (all six serines in the S/TQ-cluster were changed to aspartate as phosphomimic mutants) and xFANCI-S6A (all six serines changed to alanine as non-phosphorylated mutants) [29]. They confirmed that in ATR-phosphorylated FANCI, or any mutational combination of these three sites, xFANCI did not cause dissociation of the xI-D2 heterodimers, while xFANCI-S6D only binds weakly to FANCD2 [29], suggesting that the S6D mutation itself was influenced by the xFANCI-S6D protein to cause mis-folding. As described above, optimal I-D2 monoubiquitination requires association with DNA, and it was concluded that ATR-phosphorylation created a high affinity of FANCI and an association with FANCD2 for DNA binding, leading to a marked stimulation of monoubiquitination of FANCD2 by the FA core complex [27]. The authors performed in vitro deubiquitinination reactions where the xFANCD2-^Ubi^–FANCI-^Ubi^ complex was treated with phosphatase after ubiquitination [27]. Dephosphorylation accelerated USP1-UAF1-mediated deubiquitination, and re-phosphrylation of the xFANCD2-^Ubi^–FANCI-^Ubi^ complex by ATR kinase restored the slow rate of deubiquitination [27]. Collectively, in vitro biochemical studies suggested that ATR-mediated FANCI phosphorylation both promotes monoubiquitination and inhibits the deubiquitination of FANCD2 (Figure 3).

Cheung et al. provided a modified model using cell biological studies [28]. They found that distinct FANCI sites were phosphorylated either predominantly upstream or downstream of I-D2 monoubiquitination [28]. Ubiquitination-independent phosphorylation of FANCI S556 occurs upstream of, and promotes, FANCD2 monoubiquitination. Ubiqutination-linked phosphorylation of S559/S565 occurs predominantly downstream of monoubiquitination, and acts to inhibit FANCD2 de-ubiquitination by USP1-UAF1 and bypassed the need to deubiquitinate FANCD2 to achieve effective ICL repair [28]. Their study re-defines the activated I-D2 complex as being not only monoubiqutinated, but also phosphorylated in a ubiquitination-linked manner.

## 5. Structure of Monoubiqutinated FANCI–FANCD2 Reveals DNA Clamping

With the recent development in cryo-electron microscopy (EM) studies, significant advances have been made in the structural characterization of FA protein complexes [12,30,31,32,33]. Regarding the I-D2 complex, the previous crystal structure of a mouse I-D2 complex was solved in the absence of DNA; FANCI and FANCD2 have solenoid structures that are similar to each other [21]. The heterodimer forms a pseudo-symmetric structure, reminiscent of two antiparallel saxophone-like shapes, within which single- and double-stranded DNA binding sites were predicted. The monoubiquitin sites of mFANCD2 K559 (K561 in human) and mFANCI K522 (K523 in human) were buried in the I-D2 interface, so the ubiquitin enzymes were presumed to be difficult to access [21].

An EM structure of the monoubiqutinated I-D2 complex bound to DNA was solved by four groups from different species, i.e., human, chicken, and frog (*Xenopus*) [12,30,31,32,33]. The monoubiquitinated I-D2 complex forms a closed ring that encircles the DNA [12,30,31,32,33]. Compared with the structure of a non-ubiquitinated I-D2 complex bound to ICL DNA, which is similar to the crystal structure of a mouse I-D2 complex without DNA [31], ubiquitination induces the rotation of FANCD2, which brings FANCD2 closer to FANCI to grip the dsDNA. The ubiquitin acts as a wedge to stabilize the I-D2 closed conformation on the DNA [32]. Monoubiqutinated FANCI barely alters its conformation compared with non-ubiquitinated FANCI [33]. FANCD2 monoubiquitination changes the conformation of FANCD2, and there is a rotation of both FANCI and FANCD2, which brings their C-terminal domains toward each other [12,30,31,32,33]. An entirely new protein–protein interaction interface is created by these huge conformational changes [12,31,32]. After monubiquitnation, both of the monoubiquitin sites of FANCD2 and FANCI are exposed on the surface of the I-D2 complex. This new interface, created by monoubiquitination, rationalizes a proposed mechanism, through which deubiquitination or recruitment of the downstream DNA repair proteins, through their ubiquitin-interacting motif is controlled.

In addition, Tan et al. reported that the FANCI^-ubi^–FANCD2^-ubi^ complex formed large filament-like arrays when it was purified together with long dsDNA (plasmid DNA), but not short 60bp DNA, and was large enough to observe under an electron microscope [30]. The formed filamentous arrays may protect newly synthesized replication forks from attack by nucleases [12]. Wang et al. proposed that the FANCI^-ubi^–FANCD2^-ubi^ complex may act as a sliding DNA clamp, similar to PCNA [12,31]. The observation that FANCI^-ubi^–FANCD2^-ubi^ (and also FANCI –FANCD2^-ubi^) fully encloses DNA, and loses its substrate, specificity led to the suggestion that it might also slide (Figure 3; [26,30]). These hypotheses remain to be resolved.

## 6. Other Kinases Involved in the FA Pathway

ATR kinase strongly influences the activation of the FA pathway [34], while ATM phosphorylates many residues in FANCD2, independent of monoubiquitination, but only during the S-phase and after ionizing radiation [35,36]. DNA-PKcs could phosphorylate FANCI, but could not stimulate FANCD2 monoubiquitination [27].

It was shown that FANCI phosphorylation does not require the RAD9–RAD1-Hus1 (9-1-1) complex, the RAD17–RFC complex, or TopBP1 [19,20]. TopBP1 is the stimulator of ATR activity, as it has an ATR activation domain (AD), while the 9-1-1 complex is the recruiter of TopBP1, and the RAD17-RFC complex is the loader of the 9-1-1 complex as a junction between ssDNA and dsDNA. This mode of ATR activation in the FA pathway seems distinct and non-canonical, because, for example, Chk1 phosphorylation by ATR depends on these protein complexes [16,19,20].

Casein kinase 2 phosphorylates FANCD2 at a cluster of serines between residues 882−898, and inhibits its DNA binding and subsequent monoubiquitination [35].

In addition to FANCI, other components of the FA pathway are also known to be phosphorylated following DNA damage. The newest responsible FA FANCW/RFWD3 gene product encodes E3 ligase, which polyubiquitinates RPA and RAD51 in an ATM- and ATR-dependent manner [6,7]. FANCM is hyperphosphorylated in response to DNA damage [36,37]. However, other reports indicate that FANC proteins are phosphorylated by several kinases [16,38,39,40,41,42]. The biological significance of this phosphorylation remains to be solved.

## 7. Conclusions

FANCI phosphorylation by ATR kinase was proposed to be a molecular switch to turn on FANCD2 monoubiquitination, which is a landmark of FA pathway activation [16]. Recent studies have revealed additional evidence that S557, S560, and S565 of human FANCI residues are the direct phosphorylation target of ATR kinase, and phosphorylation of these by ATR influences the both rate of FANCD2 ubiquitination and deubiquitination to reveal how monoubiquitinated FANCD2 is maintained at DNA damage sites.

In addition to ubiquitination, deubiquitination of FANCD2 and FANCI appears to be important in the regulation of the FA pathway. There are two possible explanations for this phenomenon. One is that it prevents the retention of FANCD2 at non-repair sites in the nucleus. The other is that it allows for the completion of DNA repair [1,2,3,4,5]. A major function of FANCI^-ubi^ appears to be the protection of FANCD2^-ubi^ when it binds to DNA. FANCI dephosphorylation might be required for efficient FANCI–FANCD2 deubiquitination, as phospho-mimic mutants of FANCI prevent deubiquitination of FANCD2, both in vivo and in vitro [27,28]. In this regard, a recent paper regarding CTDP1, which is the only phosphatase that contains BRCA1 C-terminal (BRCT) domains, indicates that this is a strong candidate for this reaction [43]. CTD1 binds to FANCI, but direct evidence that CTDP1 dephosphorylates FANCI has not been reported [43].

Another significant recent finding is that the FANCI^-ubi^–FANCD2^-ubi^ complex (and the FANCI–FANCD2^-ubi^ complex) forms a closed ring that encircles double-stranded (ds) DNA, suggesting that it acts as a DNA clamp, similar to PCNA. PCNA forms a ring around DNA that slides with DNA polymerases during the DNA replication process [31]. In addition, the FANCI^-ubi^–FANCD2^-ubi^ complex forms filament-like arrays on dsDNA [30]. This reflects the recent finding that monoubiqutinated FANCD2 (possibly with FANCI) accumulates at the center of a long fragile gene locus (most of the cases are >1Mb gene length) [9,10,11]. Extensive studies are necessary in order to reveal the biological functions associated with these newly identified structures of FA proteins as a novel target for not only Fanconi anemia, but also cancer.

## Data Availability

Not applicable.
